# Decentralisation and decision space in the health sector, Karnataka, India

**DOI:** 10.1186/1753-6561-6-S5-O5

**Published:** 2012-09-28

**Authors:** Shreelata Rao Seshadri, Lakshmi Hebbare, Sandesh Kotte

**Affiliations:** 1 Ajim Premji University, Karnataka, India; 2 Centre for Global Health Research, Karnataka, India

## Introduction

Despite long-term commitment to decentralization in India, progress towards a decentralized form of governance has been rather slow. In Karnataka, despite state compliance with decentralised governance legislation from the mid-1980s, local bodies continue to suffer from inadequate power and resources. Under the National Rural Health Mission (NRHM), decentralization plays an important role in monitoring access to and quality of health services. However, after almost a decade of organized decentralisation in the health sector, its impact is still not clear, nor its ability to enhance participation of individuals/communities. There are very few studies that have tried to assess the impact of decentralization on the provision of health care services and health outcomes. There is also a lack of an analytical framework to empirically analyse its impact. Hence, there is limited evidence on the impact of decentralization on improving delivery of health care services and health outcomes worldwide.

In this regard, we undertook a study to ask: 1) does the degree of decentralization under the NRHM correlate strongly with perceived decision space of the officials of the Department of Health and local government representatives at the district level and below? 2) does the capacity of functionaries at different levels of the health system correlate with perceived decision space? 3) does greater perceived decision space by any given functionary lead to better health outcomes?

For the purpose of this research, we define decentralisation as a process, which involves shifting of power and responsibilities between tiers of government by way of various fiscal, political, and administrative instruments. We used Bossert’s analytical framework that outlines the concept of decision space, by studying the range of choice that different actors in the health system perceive as being available to them along a series of functional dimensions.

## Methods

The study builds on an initial pilot study in Tumkur district and extends it to an additional six districts of Karnataka, in a combination of developed and backward districts based on the Nanjundappa Committee report. The study includes both qualitative and quantitative data generated from questionnaires, interviews and focus group discussions at the district, sub-district and village levels.

For the quantitative analysis, we designed a decision space questionnaire administered to a range of technocrats, bureaucrats and people’s representatives at the district level and below. The questions relate to the respondents’ perceptions of power at their disposal. Qualitative data included interviews with the above-mentioned officials. Data pertaining to health outcome indicators taken from District Level Household Survey (DLHS) -3 and state NRHM program implementation plans were then analysed in relation to the perceived decision space.

## Results and discussion

The study demonstrates the relationship between perceived decision space and the effectiveness of the health system; and also provides insights into the impact of decentralization in diverse settings. Results of the study showed that overall decision space was limited among all officials at the district level. The limitation was the highest in matters relating to human resources, planning and budgeting.

Program Officers appeared to have greater decision space, and greater control within the specific boundaries of their programs. People’s representatives had little influence over decisions made by the Health Department. However, they could contribute with their own funds to specific activities such as providing additional drugs or civil works.

The results of the study suggest that the government needs to spell out in greater detail the exact activities which can be devolved to lower levels of administration; and provide the financial and operational autonomy to bring about genuine empowerment at those levels. Officials need to be trained to be able to carry out the responsibilities allocated to them and to understand better what they can and cannot do under the framework of decentralized decision making.

## Competing interests

None declared

## Funding statement

The study was funded by the Azim Premji University.

